# The Tumor-Associated Neutrophils-Related Signatures Predict Prognosis and Indicate Immune Landscape in Colorectal Carcinoma

**DOI:** 10.1155/mi/7259278

**Published:** 2025-06-05

**Authors:** Ying Wang, Yizhen Zhu, Yuling Zeng, Shuhui Gao, Youcai Tang, Ming Wei

**Affiliations:** ^1^Department of Blood Transfusion, The Second Affiliated Hospital of Zhengzhou University, Zhengzhou, China; ^2^Department of Blood Transfusion, The Fifth Affiliated Hospital of Zhengzhou University, Zhengzhou, China; ^3^Department of General Surgery, Chinese Peoples Liberation Army, Hospital of Joint Logistics Support Force 989, Luoyang, China; ^4^Department of Clinical Laboratory, Xin Yang Central Hospital, Xinyang, China; ^5^Henan Key Laboratory of Metabolic Associated Fatty Liver Disease, The Fifth Affiliated Hospital of Zhengzhou University, Zhengzhou, China

**Keywords:** colorectal carcinoma, immune landscape, prognostic signature, tumor-associated neutrophils

## Abstract

**Background:** Tumor-associated neutrophils (TANs), a major immune cell type in the tumor microenvironment (TME), are associated with antitumor or pro-tumor phenotypes. A wealth of evidence evidences demonstrated that the content of TANs has been implicated in the pathogenesis of cancer and may be ideal targets for cancer therapy. In this study, we identified TANs-related genes to construct a prognostic model and evaluated its potential as a biomarker for predicting prognosis and immunotherapeutic response in colorectal carcinoma (CRC).

**Methods:** Kaplan–Meier survival curves were used to assess the impact of TANs infiltration on patient survival. Weighted Gene Co-Expression Network Analysis (WGCNA) identified genes associated with TANs. Univariate Cox regression and least absolute shrinkage and selection operator (LASSO) regression were applied to filter TANs-related risk signatures. A prognostic model was constructed using data from The Cancer Genome Atlas (TCGA) and validated with the GSE39582 dataset. Immune cell infiltration levels and immunotherapy responses were compared between high- and low-risk groups. Functional enrichment analysis was performed to identify relevant Gene Ontology (GO) terms and pathways.

**Results:** Patients with elevated TANs levels have a low survival rate. Fourteen risk signature genes were identified, and a risk model was constructed based on these genes. Patients in the high-risk group had worse survival outcomes and improved responsiveness to immunotherapy.

**Conclusions:** This study comprehensively analyzed the role of TANs in CRC progression and developed a prognostic model based on TANs-related genes, which holds significant potential for predicting prognosis and immunotherapy outcomes in CRC patients.

## 1. Introduction

Colorectal carcinoma (CRC), with its incidence steadily increasing over the past decades, ranks as the third most prevalent cancer globally. In 2020 alone, CRC accounted for over 1.9 million new cases and 940,000 deaths, representing ~10% of all cancer diagnoses and contributing to 9.4% of global cancer-related mortality [1–3]. The multifactorial etiology of CRC, encompassing genetic predisposition, aging, lifestyle factors, environmental influences, and familial risk, has contributed to its escalating global burden, positioning it as the second leading cause of cancer-related mortality worldwide [4]. As a major subtype of gastrointestinal malignancies, CRC exhibits considerable prognostic heterogeneity, with survival outcomes largely determined by tumor stage, pathological characteristics, and biomarker profiles [5]. Despite significant advancements in diagnostic techniques and screening modalities, the majority of CRC cases are diagnosed at advanced stages (III and IV), which are associated with a poor prognosis [6]. Conventional therapeutic strategies for CRC include surgical resection, chemotherapy, radiotherapy, and targeted therapy, often used in combination to achieve optimal clinical management and improve patient outcomes [7]. However, for patients with advanced or metastatic CRC who are ineligible for surgical intervention, definitive treatment options remain limited. Consequently, CRC continues to pose a substantial public health challenge, with its pathogenesis not yet fully elucidated and prognosis remaining suboptimal [8].

The occurrence and progression of CRC are influenced by a multitude of factors within the tumor microenvironment (TME), including diverse immune cells, stromal components, and the extracellular matrix, among others. The dynamic interactions between cancer cells and immune cells play a pivotal role in shaping the biological phenotypes of cancer cells [9–11]. The infiltration of specific functional immune cell subsets in tumors has been closely correlated with prognosis and risk of postoperative recurrence in CRC patients [12]. Among these immune cells, tumor-associated neutrophils (TANs) represent a prominent immune cell population within the TME [13]. The TME can induce local accumulation of TANs through external stimulation, thereby eliciting either antitumor or pro-tumor phenotypes. On one hand, TANs can mediate antitumor effects through direct cytotoxicity and indirectly by activating adaptive immune responses [14]. On the other hand, TANs may interact with processes such as cell proliferation, angiogenesis, and immunosuppression in TME, thereby contributing to pro-tumor effects [15]. However, the prognostic significance of TANs in CRC patients remains a subject of ongoing debate [16].

Therefore, it is imperative to identify predictive markers capable of accurately forecasting disease progression in CRC and evaluating the therapeutic efficacy of chemotherapy in individual patients. In this study, we investigated the role of TANs in CRC and developed a risk score model based on TANs-related genes to assess the effectiveness of immunotherapy.

## 2. Methods

### 2.1. Data Download and Processing

The gene expression profiles and clinical information for CRC patients were obtained from two databases: The Cancer Genome Atlas GDC database (TCGA-GDC) (https://portal.gdc.cancer.gov/) and Gene Expression Omnibus (GEO) database (GSE39582). Only CRC patients with complete survival information were included in the analysis. The TCGA-CRC dataset, consisting of 541 CRC patients with comprehensive clinical information, was selected as the training set. Additionally, survival data on progression-free survival (PFS) and disease-specific survival (DSS) for TCGA-CRC were retrieved from UCSC-Xena platform (https://xena.ucsc.edu/). The GSE39582 dataset comprises sequencing data from a total of 585 patients. Following the removal of 19 normal control samples and 9 samples with missing overall survival (OS) times and survival times of less than 30 days, a total of 557 samples were included in this study as the validation dataset. Data processing and result visualization were performed using R software version 4.1.3 and Perl version 5.30.0.

### 2.2. Neutrophils Fraction and Related Survival Analyses

Currently, a variety of computational algorithms are available for quantifying immune cell infiltration in tumors, and the integration of multiple algorithms can enhance the reliability of the obtained results. MCPcounte is a quantitative algorithm that estimates immune cell infiltration levels based on marker genes. xCell utilizes a single-sample gene set enrichment analysis (ssGSEA)-based method to generate enrichment scores reflecting immune cell abundance. Meanwhile, the CIBERSORTx algorithm deconvolutes immune cell proportions by analyzing the expression profiles of 22 distinct immune cell phenotypes. In our study, the infiltrating fraction of neutrophils in each CRC sample was estimated using the three algorithms mentioned above (MCPcounter, CIBERSORTx, and xCell). After merging with the survival data, the CRC samples were divided into high- and low-infiltrating groups based on the optimal cutoff value determined using the “survminer” and “survival” package. The Kaplan–Meier method was employed to compare the discrepancy in OS between the low- and high-level infiltrating neutrophils.

### 2.3. Identification TANs-Related Genes

The TANs-related genes were identified from a gene module that exhibited correlation with the infiltrating level of neutrophils using Weighted Gene Co-expression Network Analysis (WGCNA). WGCNA, employed to investigate the association between traits and gene expression, detected clusters with highly correlated with neutrophil content [17]. Firstly, genes with significant expression were included in the analysis. To assess the relevance of all samples, CRC samples were clustered using Pearson correlation coefficient calculation in both TCGA-CRC and GSE39582 datasets. When the soft threshold power *β* = 3 or 4, the scale-free topological network based on TCGA-CRC and GSE39582 data exhibited high connectivity, respectively. In addition, a hierarchical clustering gene dendrogram of the TOM matrix was constructed, excluding gene dendrograms containing less than 30 genes. Highly similar gene dendrograms were merged into modules (cutoff threshold < 0.2). Subsequently, we calculated the correlation between gene modules and neutrophil contents, selecting the gene modules that were closely related to all three methods of estimating immune infiltration. Finally, we intersected modular genes obtained from the TCGA-CRC dataset with those from GSE39582 dataset to identify TANs-related genes.

### 2.4. Construction and Validation TANs-Related Genes Prognostic Model

Univariate Cox proportional hazard regression (uniCox) analysis was applied to filter prognostic genes among TANs-related genes. In order to elucidate the potential relationship and prognostic value of these uniCox gene signatures, a prognostic interaction network was constructed. Next, least absolute shrinkage and selection operator (LASSO) regression was utilized to narrow down the range of prognostic TANs-related genes. Following the uniCox and LASSO analyses, 14 risk signatures were identified for establishing the prognostic model. To further elucidate the role of these risk genes in neutrophils, we analyzed the correlation between risk signatures and TANs-related genes in cancer carcinogenesis and metastasis [18]. The risk score for each CRC patient was calculated using the formula: Risk score=∑iCoeffient (TANs-related genei) × Expression (TANs-related genei). Based on the median risk score, the CRC patients were categorized into high-risk and low-risk groups. Then, the Kaplan–Meier survival curve evaluating the prognostic value of the model was generated by R packages “survival” and “survminer” in train dataset (TCGA-CRC). Furthermore, to validate its feasibility, Kaplan–Meier survival curves were also plotted for different stages of TCGA-CRC patients as well as in an independent test dataset (GSE39582 dataset). Receiver operating characteristic (ROC) curves and area under the curve (AUC) representing sensitivity and specificity of this model at 1-, 3-, and 5-year survival time points were plotted using R package “timeROC.” Univariate and multivariable regression analyses explored independent prognostic factors among various clinical characteristics including risk score, age, gender, and stage.

### 2.5. Establishment and Assessment Nomogram Model

To quantify the contribution of each indicator in the risk model, we constructed a Nomogram to predict the OS time of patients. The calibration curve was employed to assess the concordance between predicted and observed OS values in the nomogram model. Decision curve analysis (DCA) was utilized to evaluate the net benefit of an intervention for patients based on the prediction model. ROC analysis was applied to assess the predictive accuracy of the model.

### 2.6. Analyses of Immune Cells Infiltration and Immunotherapy

TME affects the prognosis and immunotherapy response of patients. Immune score, stromal score, and tumor purity were utilized to evaluate the proportions of immune cells, stromal cells, and tumor cells, respectively. The “ESTIMATE” and “limma” R packages were employed to calculate these scores. Additionally, the relation between immune infiltration statuses and risk score was compared with the “CIBERSORT,” “reshape2,” “ggplot2,” and “ggpubr” R package. Next, we chose some immune checkpoint genes (Supporting Information Table S1) to probe into the discrepancy of immunotherapy response between the high-risk group and the low-risk group. Additionally, microsatellite instability (MSI) and tumor mutation burden (TMB), which are common biomarkers distinguishing responders from non-responders to immunotherapy, were also considered [19].

### 2.7. GSEA

The GSEA and ssGSEA were conducted using GSEA and R software to elucidate the underlying mechanisms contributing to differential performance between low- and high-risk groups.

### 2.8. Statistical Analysis

Statistical analyses and data visualization were conducted using (version 4.1.2) and Perl (version 5.30.0), with aforementioned R packages utilized for statistical computations. The Wilcoxon test was employed to compare differences between two risk groups, while survival analyses were performed using the Kaplan–Meier method with log-rank test. Univariate Cox and LASSO regressions were used to establish a prognostic risk model for CRC patients, and correlation analysis was conducted using Pearson's method. The ROC curve and AUC were used to evaluate the model's sensitivity and specificity. Univariate and multivariate regression analyses, along with a nomogram, were employed to assess prognosis. *p* < 0.05 was considered significant.

## 3. Results

### 3.1. The Content of TANs Affects Overall Survival of Colorectal Cancer Patients

**Figure 1:**
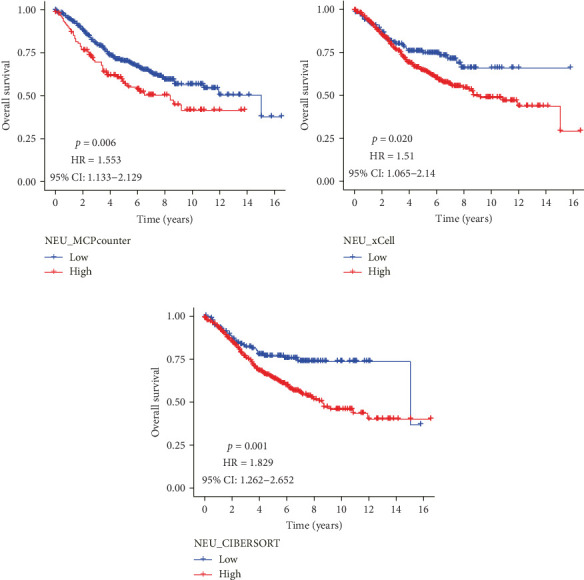
Kaplan–Meier survival curves showed difference in the overall survival between groups with the high-content group and the low-content group of TANs using MCPcounter (A), xCell (B), and CIBERSOR (C) algorithm based on GSE39582 data. TANs, tumor-associated neutrophils.

Analysis of CRC TME using the MCPcounter algorithm revealed that higher TANs infiltration levels correlated with shorter OS (Figure 1A). This negative prognostic association was consistently replicated by two additional computational approaches: xCell (Figure 1B) and CIBERSORTx (Figure 1C). Collectively, these findings establish TANs as critical mediators in CRC pathogenesis and disease progression.

### 3.2. Acquisition of TANs-Associated Genes

**Figure 2:**
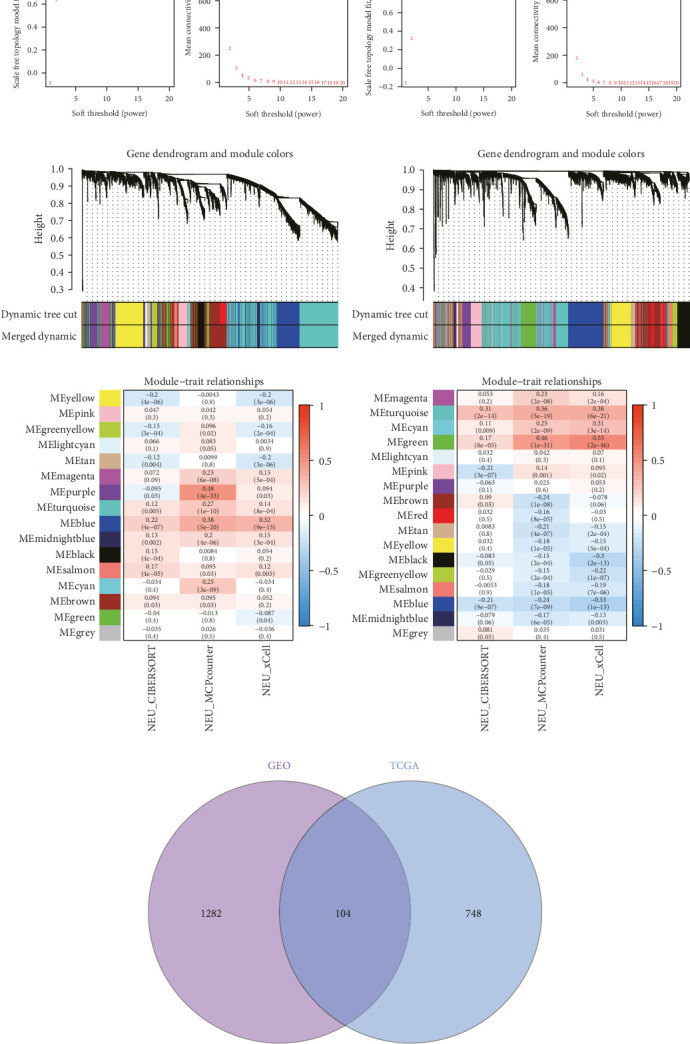
Acquisition of TANs associated genes using WGCNA. Screening for suitable soft thresholds for TCGA (A) and GEO data (B), respectively. The cluster dendrogram with the gene modules and module merging for TCGA (C) and GEO data (D), respectively. The correlation between gene modules and TANs using three algorithms based on TCGA (E) and GSE39582 data (F), respectively. (G) Venn diagram displaying the overlaps of modular genes from the TCGA-CRC dataset and GSE39582 dataset. GEO, Gene Expression Omnibus; TANs, tumor-associated neutrophils; TCGA, The Cancer Genome Atlas; WGCNA, weighted gene co-expression network analysis.

To further illustrate the role of TANs in CRC, TANs-related genes were filtered using the WGANA algorithm. Subsequently, all significant expressed genes were divided into 16 gene modules on the basis of the optimal soft-threshold power: *β* = 3 in TCGA-CRC dataset (Figure 2A,C). In GSE39582 dataset, a value of 4 was determined as the optimal *β* value (Figure 2B), resulting in recognition of 17 gene modules (Figure 2D). Notably, both MEblue and MEturquoise gene modules exhibited a positive association with TANs content (Figure 2E,F). In order to enhance the precision of TANs-related genes, we identified 104 candidate risk genes by intersecting the two gene modules from TCGA-CRC and GSE39582 datasets (Figure 2G). Furthermore, Gene Ontology (GO) and KEGG enrichment analyses were performed to gain insights into these candidate genes' functions. The results of GO enrichment analyses revealed that these risk genes were correlated with cytokine activity, chemokine receptor binding, and immune response processes (Supporting Information Figure S1A). The KEGG pathway analysis suggested that the genes were involved in the IL-17 signaling, TNF signaling, NF-kappa B signaling, transcriptional misregulation in the cancer pathway (Supporting Information Figure S1B).

### 3.3. Construction and Validation TANs-Associated Prognostic Signature

**Figure 3:**
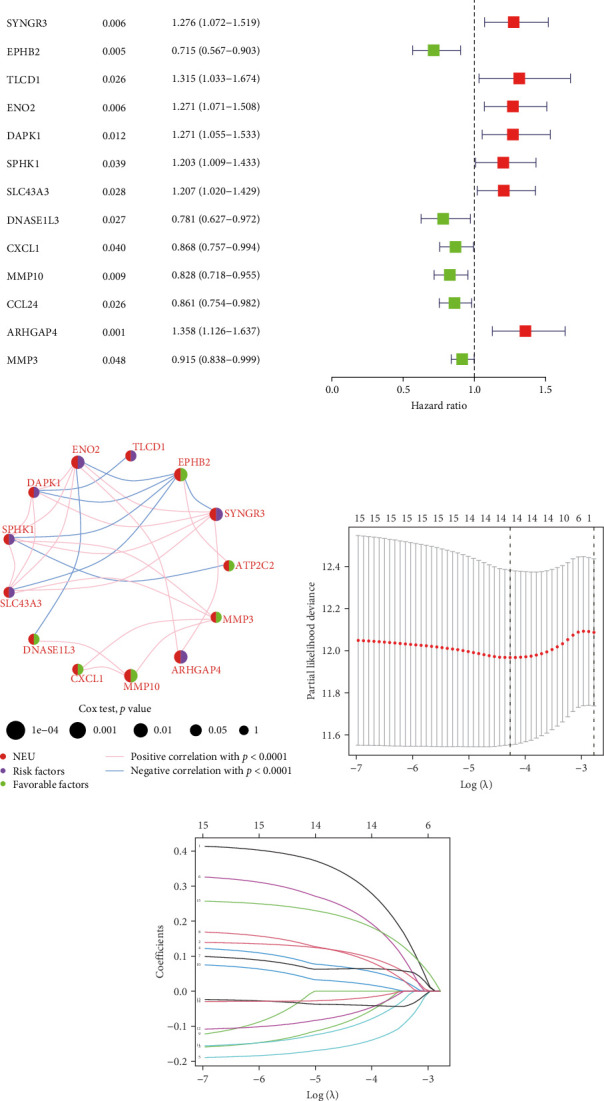
Development of TANs-associated prognostic signature. (A) Univariate Cox regression identified 16 neutrophil-related genes with significant survival associations. (B) Protein–protein interaction network constructed from prognostic genes, highlighting three hub nodes. (C) Ten-fold cross-validation curve determining optimal *λ* value (*λ* = 0.032) for LASSO regularization. (D) LASSO coefficient trajectories showing feature selection process across penalty parameters. LASSO, least absolute shrinkage and selection operator; TANs, tumor-associated neutrophils.

**Figure 4:**
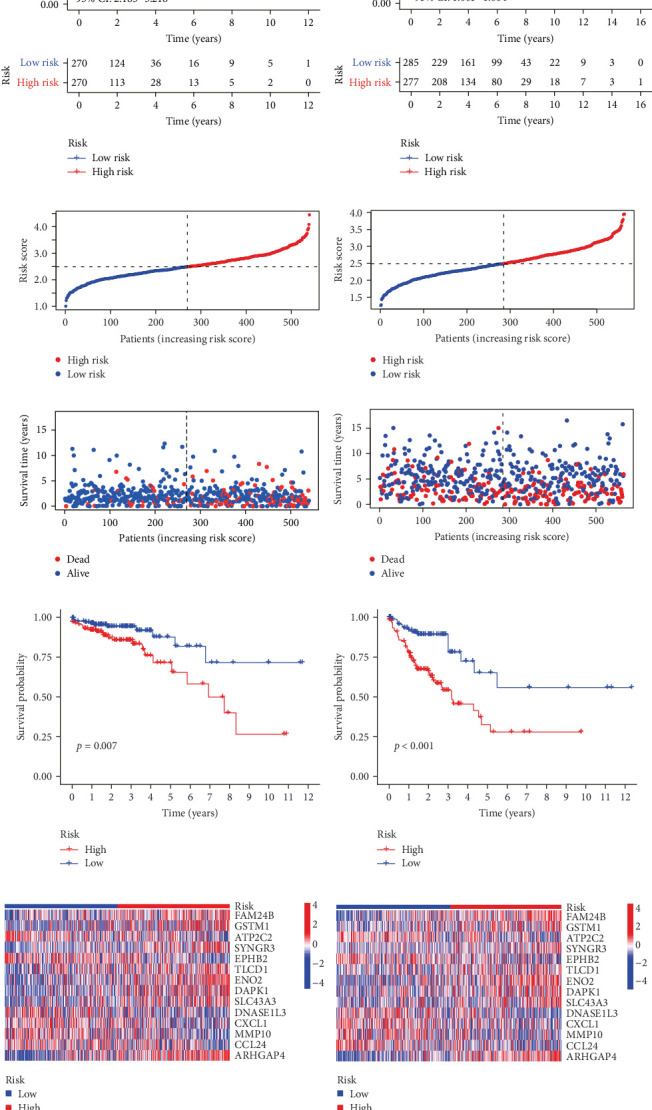
Construction and validation of TANs-associated prognostic signature in colorectal cancer. (A, B) Kaplan–Meier survival analysis comparing high- versus low-risk groups in TCGA (A) and GSE39582 (B) cohorts. (C, D) Risk score distribution patterns across patients in TCGA (C) and GSE39582 (D) datasets. (E, F) Mortality rate escalation with increasing risk scores in TCGA (E) and GSE39582 (F) populations. (G, H) Stage-stratified survival analysis for early-stage (I–II, G) and advanced-stage (III–IV, H) TCGA-CRC subgroups. (I, J) Differential expression heatmap of neutrophil-related genes between risk groups in TCGA (I) and GSE39582 (J) cohorts. CRC, colorectal carcinoma; TANs, tumor-associated neutrophils; TCGA, The Cancer Genome Atlas.

Initial screening of 104 candidate genes through univariate Cox analysis identified 16 prognostic signatures (Figure 3A). Network analysis delineated functional interactions among these biomarkers (Figure 3B). Subsequent LASSO regression refinement further optimized the model, yielding 14 core prognostic genes: FAM24B, GSTM1, ATP2C2, SYNGR3, EPHB2, TLCD1, ENO2, DAPK1, SLC43A3, DNASE1L3, CXCL1, MMP10, CCL24, and ARHGAP4 (Figure 3C,D). Furthermore, the risk signature genes demonstrated significant associations with TANs-regulated genes critically involved in tumorigenesis and metastatic progression (Supporting Information Figure S2). Patients were categorized into high- and low-risk groups across training and test cohorts using validated risk scores. Survival curves revealed significantly poorer overall survival in high-risk patients versus low-risk counterparts in both training (Figure 4A,C) and validation cohorts (Figure 4B,D). The TANs-associated risk signature showed significantly elevated expression in high-risk groups compared to low-risk groups in training (Figure 4E) and validation datasets (Figure 4F). Stratification analysis confirmed this prognostic pattern in both early-stage (I–II) and advanced-stage (III–IV) TCGA-CRC cohorts (Figure 4G,H). Differential expression patterns of TANs-related genes between risk groups were visualized through heatmaps in TCGA-CRC (Figure 4I) and GSE39582 (Figure 4J) cohorts.

### 3.4. Evaluation of TANs-Associated Prognostic Signature Performance

**Figure 5:**
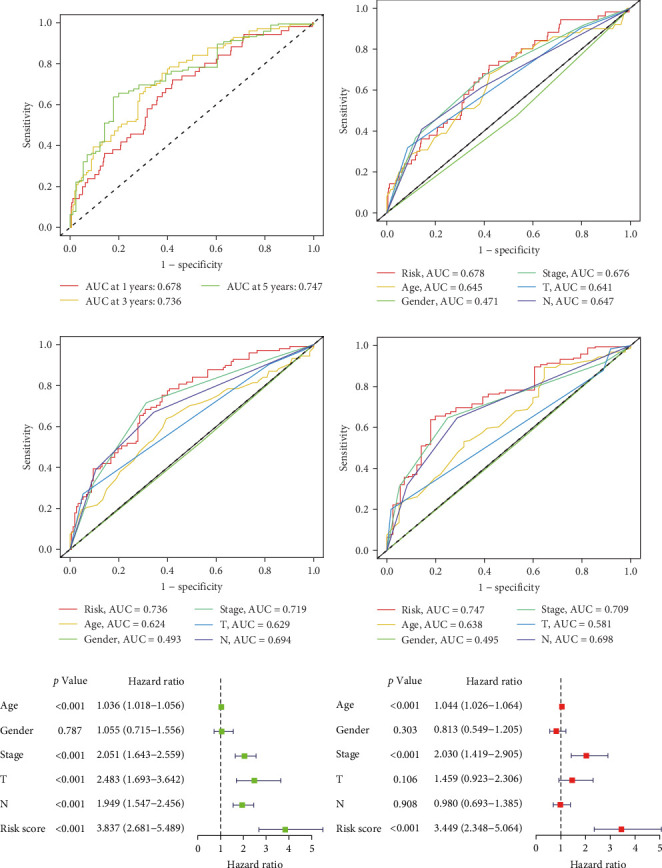
Evaluation of TANs-associated prognostic signature performance. (A) Time-dependent ROC curves demonstrating 1-, 3-, and 5-year overall survival (OS) predictive accuracy. (B–D) Comparative ROC analyses between the TANs signature and conventional clinical parameters (age, gender, TNM stage) at 1-, 3-, and 5-year intervals. (E, F) Forest plot of univariate Cox regression (E) and multivariate Cox regression (F) confirming independent prognostic value. ROC, receiver operating characteristic; TANs, tumor-associated neutrophils.

To evaluate the prognostic capacity of our model, we performed time-dependent ROC analysis, multivariable regression, and nomogram construction. The risk signature exhibited temporally discriminative performance, with 5-year survival prediction achieving the highest AUC (0.75, 95% confidence interval [CI] 0.69–0.81), followed by 3-year (AUC = 0.74, 95% CI 0.68–0.80) and 1-year (AUC = 0.68, 95% CI 0.61–0.75) predictions (Figure 5A). Notably, the signature consistently surpassed conventional clinical parameters (age, gender, TNM stage) in prognostic accuracy across all temporal endpoints (Figure 5B–D). Multivariable Cox regression confirmed the risk score as an independent prognostic determinant (univariate HR = 3.872, 95% CI 2.681–5.489; multivariate HR = 3.449, 95% CI 2.348–5.064; Figure 5E,F).

### 3.5. Construction and Evaluation of a Nomogram

**Figure 6:**
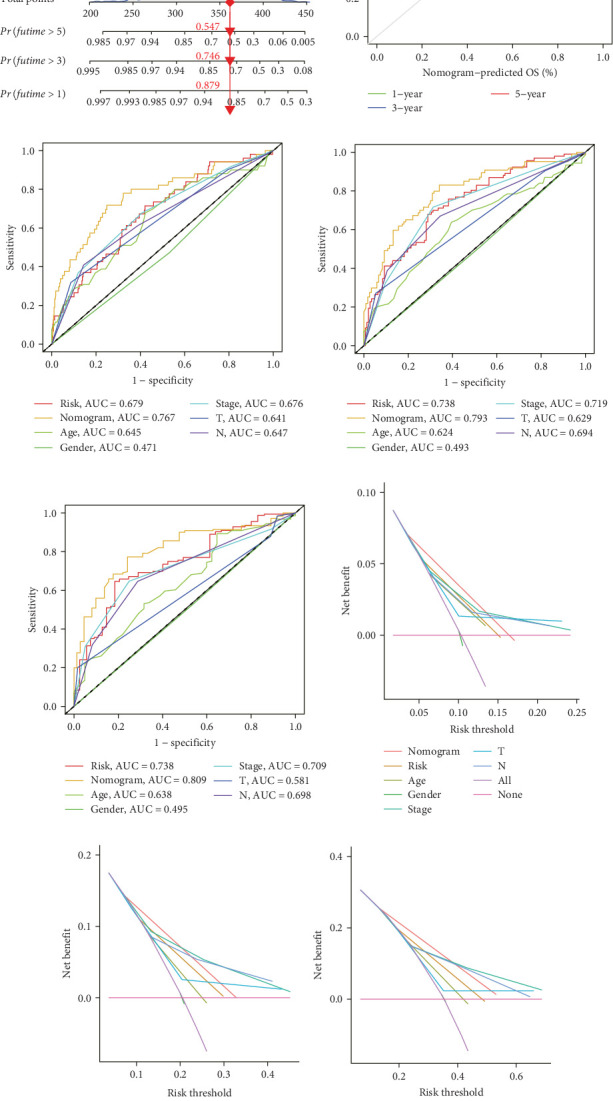
Construction and validation of a nomogram. (A) Nomogram for predicting the 1-, 3-, and 5-year OS of CRC patients. (B) Calibration curves of the nomogram-predicted and observed 1-, 3-, and 5-year survival. (C–E) The time-dependent ROC curves of the nomograms compared for 1-, 3-, and 5-year OS in CRC, respectively. (F–H) The decision curve analysis of the nomograms compared for 1-, 3-, and 5-year OS in CRC, respectively. CRC, colorectal carcinoma; ROC, receiver operating characteristic.

The clinical nomogram integrating risk scores with TNM staging demonstrated precise calibration for 1–, 3-, and 5-year survival predictions (Figure 6A), with close alignment between predicted and observed outcomes (Figure 6B). Time-dependent ROC analysis showed the combined model outperformed standalone risk-score or clinical-feature models, achieving higher AUC values at 1-year (0.767 vs 0.679), 3-year (0.793 vs 0.738), and 5-year (0.809 vs 0.738) intervals (Figure 6C–E). Additionally, the nomogram model yielded superior clinical net benefit in forecasting prognosis compared to alternative models as evidenced by DCA (Figure 6F–H).

### 3.6. Analyses of Immune Cells Infiltration and Immunotherapy

**Figure 7:**
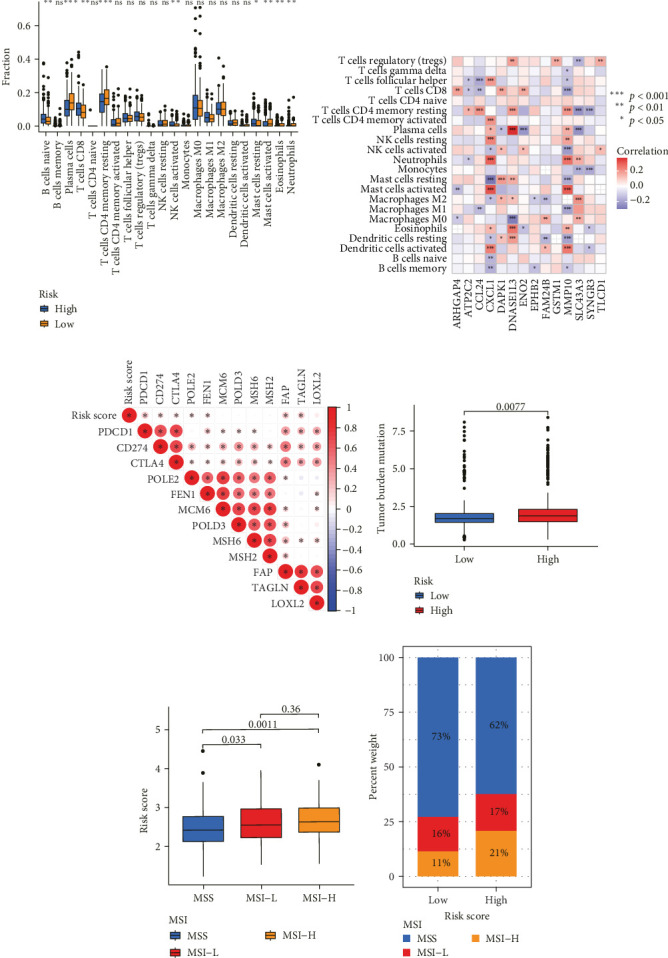
The role of TANs-related genes prognosis model in immunotherapy. (A) The difference expression of infiltrating immune cell between high-risk group and the low-risk group. (B) The association between the infiltrating immune cell and risk signatures. (C) Relationships between risk score and immune checkpoints. (D) Relationships between risk score and TMB. (E, F) Relationships between risk score and MSI. MSI, microsatellite instability; TANs, tumor-associated neutrophils; TMB, tumor mutation burden.

Correlation analysis between risk score and immune cell content signified a different TME characterized in high- and low-risk groups. The high-risk patients exhibited significant enrichment of CD8^+^T cell, activated NK cells, and resting mast cells, while concurrently showing depletion of plasma cells, resting CD4^+^ memory T cells, activated mast cells, eosinophils (Figure 7A). Moreover, the risk signatures (CXCL1 and MMP10) showed significant correlations with multiple types of immune cell infiltration (Figure 7B). Notably, the risk score demonstrated strong positive correlations with key immune checkpoint regulators, including PD-1 (PDCD1), PD-L1 (CD274), CTLA-4, FAP, and TAGLN (*P* < 0.05) (Figure 7C). The response of immune checkpoint inhibitors was evaluated by MSI and TMB. Compared with the low-risk group, high-risk patients had higher TMB and a higher proportion of MSI-high (MSI-H) (Figure 7D–F).

### 3.7. Function Enrichment Analysis

**Figure 8:**
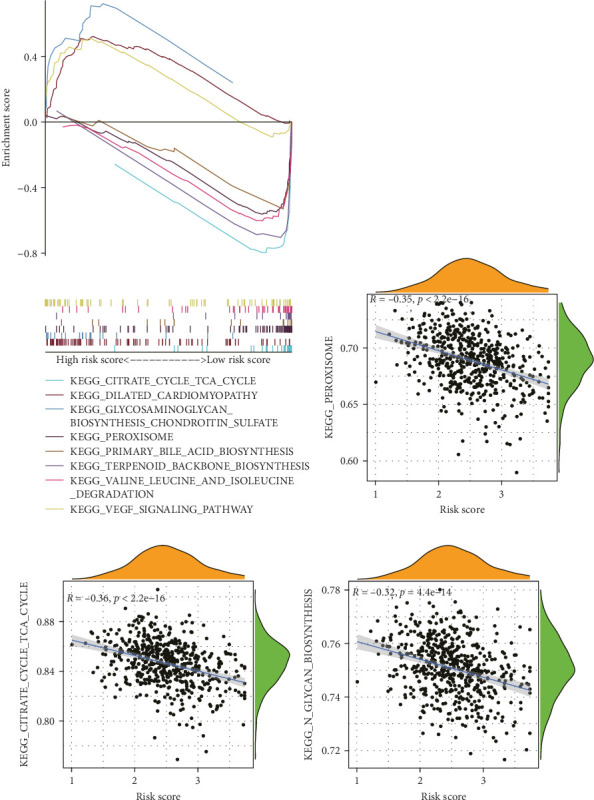
Gene set enrichment analysis of TANs-related signature in high- and low-risk groups. (A) Gene set enrichment analysis. (B–D) Risk score by ssGSEA. ssGSEA, single sample gene set enrichment analysis; TANs, tumor-associated neutrophils.

To elucidate the direction, we performed enrichment analysis to investigate the pathways differentially enriched in the high-risk and low-risk groups. The high-risk score group exhibited enrichment of glycosaminoglycan biosynthesis, chondroitin, and VEGF signaling pathway. Conversely, the low-risk score group showed enrichment of citrate cycle TCA cycle, peroxisome, valine leucine, and isoleucine degradation pathway (Figure 8A). Additionally, ssGSEA analysis revealed a significant negative correlation between risk score and citrate cycle TCA cycle, peroxisome, as well as N glycan biosynthesis pathway (Figure 8B–D).

## 4. Discussion

With the recent changes in diet and lifestyle in recent decades, the incidence rate and mortality of CRC increased progressively in developing countries [20, 21]. The inherent heterogeneity of CRC necessitates tailored treatment strategies based on the extent of tumor invasion, often resulting in inconsistent outcomes [22, 23]. The excavation of operable targets and the realization of precision therapy are crucial for improving survival rates in CRC patients. TANs, as “assistants” of immune checkpoint inhibitors (ICIs) treatment, have been implicated as an important prognosis factor in multiple tumors, including CRC. However, the relationship between tumor-infiltrating neutrophils and CRC prognosis remains controversial [24]. In this study, we demonstrated that elevated TANs levels are associated with unfavorable prognosis in CRC patients. It is similarly that high neutrophil-to-lymphocyte ratio (NLR) levels have been associated with shorter PFS and disease-free survival [25]. Furthermore, we developed a risk score model based on TANs-related genes, which demonstrated superior prognostic value and potential treatment efficacy for CRC.

A plethora of studies have demonstrated the pivotal role of TANs in promoting tumor inflammation and contributing to tumorigenesis [26], progression, and prognosis in breast, hepatocellular [27] and gastric cancers. Mechanistically, the mutual crosstalk between TANs and tumor cells induces various changes in tumor cells that are favorable to metastasis. AGR2, a homologue of *Xenopus laevis* anterior gradient protein 2 (XAG-2), promotes tumorigenesis and progression [28], and TANs are the main cell type secreting AGR2 in the CRC TME. AGR2 secreted by TAN binds to CD98hc-xCT on CRC cells, enhances the activity of xCT, activates related signaling pathways, and promotes cancer cell migration. Transforming growth factor-*β*1 (TGF-*β*1) produced by CRC cells promotes the migration of peripheral blood neutrophils (PBNs), giving PBNs characteristics similar to TANs, which further promotes the secretion of AGR2, forming a positive feedback loop and exacerbating tumor progression. The number of infiltrating AGR2^+^ TANs also correlates with the patient's prognosis, with the higher the number, the worse the prognosis [29]. In addition, TANs promoted the angiogenesis and accelerated liver metastases of CRC [18]. TANs were highly expressed lysyl oxidase-like 4 in CRC that may be the key factor that leads to the resistance to antiangiogenic therapy [30]. Seubert et al. [31] proposed that SDF-1-dependent TANs recruitment to the liver is crucial for TIMP-1-induced premetastatic. Building upon the theoretical foundation that TANs were an unfavorable prognosis marker for CRC, we further exploited TANs-associated genes by WGCNA and established a risk score model included 14 genes to evaluate the prognosis of CRC. In comparison to other clinical characteristics such as stage, the risk score model as an independent prognostic factor obtained better prognostic value for CRC.

Cancer immunotherapies, including adoptive cell transfer and ICIs, was based on manipulating the immune system to reactivate the antitumor immune response and overcome the pathways leading to escape. Recently, it has obtained durable clinical responses, but their efficacies vary, and only subsets of cancer patients can benefit from them [32, 33]. Therefore, there is an urgent clinical need for predictive markers to stratify patients and identify those who will benefit from immune checkpoint inhibitor therapy. TANs play a critical role in regulating the TME and the anti-tumor immune response, and can influence the efficacy of ICIs through multiple pathways [34]: First, TANs inhibit T-cell function and recruit immunosuppressive regulatory T cells (Tregs); Second, TANs promote the formation of immature vascular networks that hinder the extravasation and recruitment of immune cells into tumor tissue and trigger immune rejection; Third, neutrophil accumulation in tumor tissues also limits the efficacy of ICIs, for example, lack of the tumor suppressor genes LKB1/STK11 in lung cancer recruits more tumor-promoting TANs, leading to resistance to immune checkpoint blockade therapy [35]. Therefore, based on these studies, we investigated the potential of TAN-based risk models in predicting immunotherapy efficacy. The vast majority of CRC patients are in MSS, which is consistent with the finding by Koopman et al. that “~15% of CRC patients have MSI” [36]. MSI is considered a favorable prognostic factor and a strong negative predictor of efficacy of fluoropyrimidines in stage II colon cancer [37], whereas it may have a small negative impact on survival in the metastatic setting. Thus, there are conflicting data on MSI in predicting prognosis: Goldstein's retrospective analysis of the difference in OS between MSI and MSS patients was not statistically significant [38], whereas Sinicrope et al. found a more favorable impact of MSI status on OS compared to MSS [37]. However, patients with CRC tumors with MSI have an improved response to PD-1 inhibitor therapy [39, 40], which is now well established in research. In CRC, the combination of PD-1 inhibitor nivolumab, with or without ipilimumab, has demonstrated promising response rates and improved survival outcomes in patients with mismatch repair deficiency (dMMR) or high levels of MSI-H metastatic CRC [41, 42]. It was found that the MSI-H cohort had higher CD8^+^ T cells and increased presence of NK cells compared to the MSS group and may have a more active tumor immune response. In our study, the MSI-H cohort had higher risk scores and higher levels of CD8^+^ T cells and NK cell immune infiltration in the TME of patients in the high-risk group than in the low-risk group; therefore, patients with MSI-H in the high-risk group may have a higher rate of immune response to PD-1 inhibitors. In addition, elevated neutrophil levels have been found to be an independent predictor of rapid disease progression in patients treated with ICIs [43]. Therefore, the risk model established based on neutrophil-related genes has great potential in predicting the efficacy of ICIs. Our model has preliminarily shown that high-risk patients have a higher TMB and a higher proportion of MSI-H. In the next step, our research group plans to extensively collect clinical data of CRC patients, including survival time, ICIs treatment regimens, and response rates. Additionally, we will detect the expression levels of MSI markers in patients to comprehensively evaluate the performance of this risk model in predicting the efficacy of ICIs.

## 5. Conclusions

In conclusion, our findings unveil the detrimental impact of TANs on CRC prognosis and establish a risk score model based on TANs-related genes, which demonstrates potential value in stratifying CRC patients for their likelihood of responding to immunotherapy.

## Data Availability

The original contributions presented in the study are included in the article/Supporting Information. Further inquiries can be directed to the corresponding authors. Besides, the data that support the finding of this study are openly available in https://portal.gdc.cancer.gov/ and https://www.ncbi.nlm.nih.gov/geo/.

## Supporting Information

Supporting Information 1 Figure S1: KEGG and GO enrichment analysis in 104 TANs-related genes. (A) GO enrichment and (B) KEGG signaling pathway analysis for 104 TANs-related genes.

Supporting Information 2 Figure S2: Fourteen risk signatures correlated with TAN-related genes in cancer carcinogenesis and metastasis.

Supporting Information 3 Table S1: Immune checkpoint genes, to probe into the discrepancy of immunotherapy response between high-risk group and low-risk group.
