# Supplementary figures and images for: The Tumor-Associated Neutrophils-Related Signatures Predict Prognosis and Indicate Immune Landscape in Colorectal Carcinoma
Source: Mediators Inflamm. 2025 Jun 5;2025:7259278. doi: 10.1155/mi/7259278 (PMC12162164; doi:10.1155/mi/7259278)

A

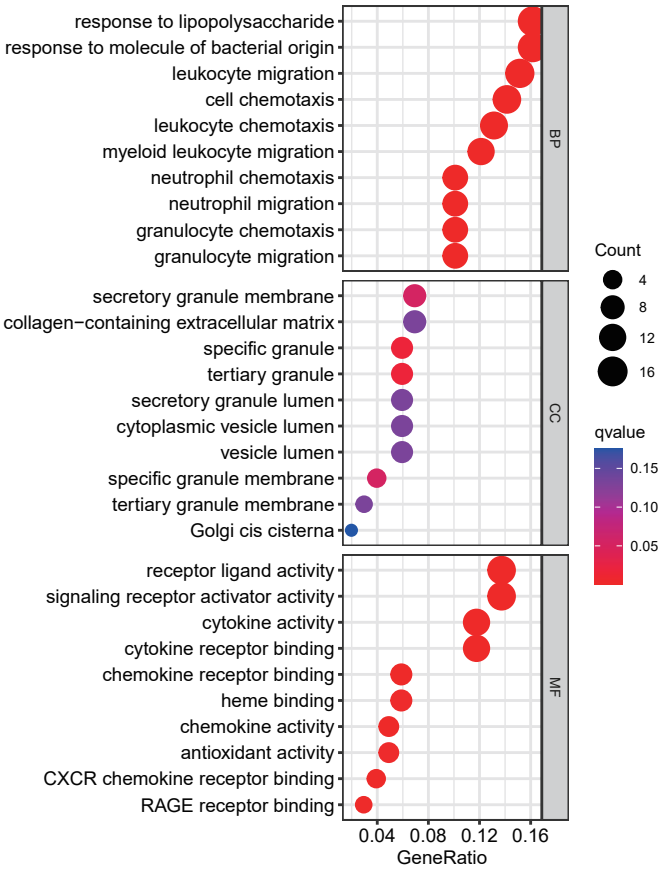

B

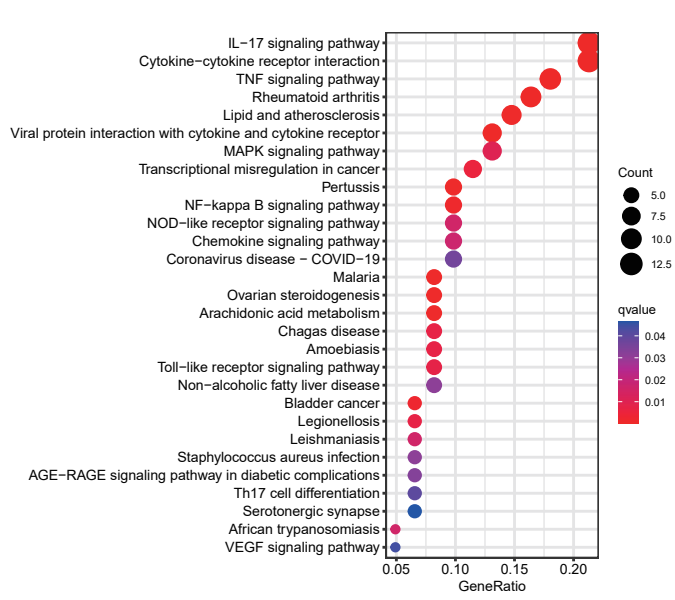

Supplement: Supporting Information 1 — Figure S1: KEGG and GO enrichment analysis in 104 TANs-related genes. (A) GO enrichment and (B) KEGG signaling pathway analysis for 104 TANs-related genes. [file 7259278.f1.pdf]

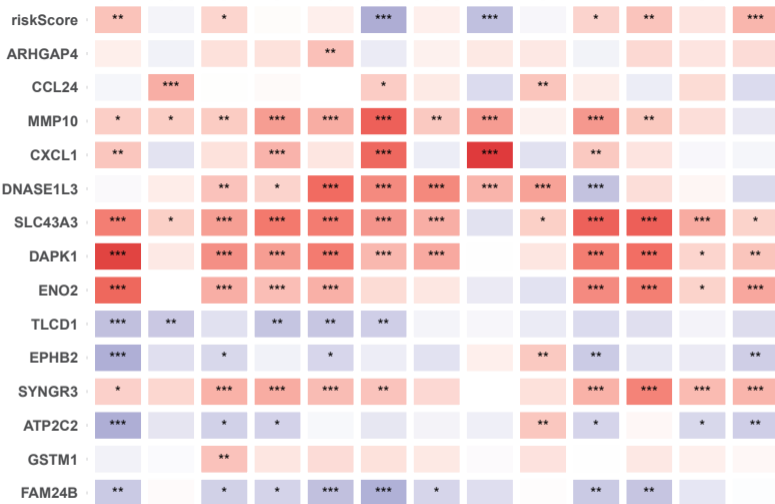

\*\*\* p<0.001

\*\* p<0.01

\* p<0.05

Correlation

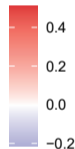

Supplement: Supporting Information 2 — Figure S2: Fourteen risk signatures correlated with TAN-related genes in cancer carcinogenesis and metastasis. [file 7259278.f2.pdf]
