# Supplementary material for: The Tumor-Associated Neutrophils-Related Signatures Predict Prognosis and Indicate Immune Landscape in Colorectal Carcinoma
Source: Mediators Inflamm. 2025 Jun 5;2025:7259278. doi: 10.1155/mi/7259278 (PMC12162164; doi:10.1155/mi/7259278)
Supplement: Supporting Information 3 — Table S1: Immune checkpoint genes, to probe into the discrepancy of immunotherapy response between high-risk group and low-risk group. [file 7259278.f3.docx]

| immune checkpoint genes |
| --- |
| PDCD1 |
| CD274 |
| CTLA4 |
| POLE2 |
| FEN1 |
| MCM6 |
| POLD3 |
| MSH6 |
| MSH2 |
| FAP |
| TAGLN |
| LOXL2 |

immune checkpoint genes: to probe into the discrepancy of immunotherapy response between high-risk group and low-risk group.
